# Providing Human Support for the Use of Digital Mental Health Interventions: Systematic Meta-review

**DOI:** 10.2196/42864

**Published:** 2023-02-06

**Authors:** Alexandra Werntz, Selen Amado, Megyn Jasman, Ariel Ervin, Jean E Rhodes

**Affiliations:** 1 Center for Evidence-Based Mentoring University of Massachusetts Boston Boston, MA United States

**Keywords:** digital mental health interventions, human support, supportive accountability, systematic meta-review

## Abstract

**Background:**

Digital mental health interventions (DMHIs) have been increasingly deployed to bridge gaps in mental health care, particularly given their promising efficacy. Nevertheless, attrition among DMHI users remains high. In response, human support has been studied as a means of improving retention to and outcomes of DMHIs. Although a growing number of studies and meta-analyses have investigated the effects of human support for DMHIs on mental health outcomes, systematic empirical evidence of its effectiveness across mental health domains remains scant.

**Objective:**

We aimed to summarize the results of meta-analyses of human support versus no support for DMHI use across various outcome domains, participant samples, and support providers.

**Methods:**

We conducted a systematic meta-review of meta-analyses, comparing the effects of human support with those of no support for DMHI use, with the goal of qualitatively summarizing data across various outcome domains, participant samples, and support providers. We used MEDLINE, PubMed, and PsycINFO electronic databases. Articles were included if the study had a quantitative meta-analysis study design; the intervention targeted mental health symptoms and was delivered via a technology platform (excluding person-delivered interventions mediated through telehealth, text messages, or social media); the outcome variables included mental health symptoms such as anxiety, depression, stress, posttraumatic stress disorder symptoms, or a number of these symptoms together; and the study included quantitative comparisons of outcomes in which human support versus those when no or minimal human support was provided.

**Results:**

The results of 31 meta-analyses (505 unique primary studies) were analyzed. The meta-analyses reported 45 effect sizes; almost half (n=22, 48%) of them showed that human-supported DMHIs were significantly more effective than unsupported DMHIs. A total of 9% (4/45) of effect sizes showed that unsupported DMHIs were significantly more effective. No clear patterns of results emerged regarding the efficacy of human support for the outcomes assessed (including anxiety, depression, posttraumatic stress disorder, stress, and multiple outcomes). Human-supported DMHIs may be more effective than unsupported DMHIs for individuals with elevated mental health symptoms. There were no clear results regarding the type of training for those providing support.

**Conclusions:**

Our findings highlight the potential of human support in improving the effects of DMHIs. Specifically, evidence emerged for stronger effects of human support for individuals with greater symptom severity. There was considerable heterogeneity across meta-analyses in the level of detail regarding the nature of the interventions, population served, and support delivered, making it difficult to draw strong conclusions regarding the circumstances under which human support is most effective. Future research should emphasize reporting detailed descriptions of sample and intervention characteristics and describe the mechanism through which they believe the coach will be most useful for the DMHI.

## Introduction

### Background

Over the past 2 decades, a growing number of digital mental health interventions (DMHIs) have leveraged technology to address common mental health concerns, including anxiety, depression, obsessive-compulsive disorder, and suicidal ideation [1-3]. Research continuously supports the efficacy of several DMHIs [4-6], especially those that use cognitive behavioral therapy principles and include methods to cope with stress, such as journaling or tracking thoughts, feelings, and behaviors [1,7]. DMHIs can include mental health mobile apps (MHAs) and computer-based interventions [8-10], which deliver on-demand support ranging from behavioral strategies (eg, self-monitoring) to more complex therapeutic approaches (eg, cognitive behavioral therapy) [8-13]. Clinician-delivered interventions, such as an hour of psychotherapy or a dose of medication, are costly and *consumable* (ie, once delivered to 1 client, they cannot be used to treat another), whereas DMHIs are *nonconsumable*, in that they can be delivered with high fidelity multiple times [14,15]. Human-supported DMHIs have the potential to offer a cost-effective, sustainable way of scaling access to high-quality interventions.

In addition to their scalability and affordability, DMHIs often include dynamic features such as games, animation, and badging [16-19]; provide data collection features to evaluate efforts and present in-time data dashboards; and can readily incorporate new advances in research and practice [20,21]. DMHIs can also reduce stigma and provide a sense of privacy that typical therapeutic practices may not be able to offer, especially for underserved populations. Given these and other benefits, DMHIs may be able to dramatically extend the reach of evidence-based care and reduce the global burden of mental health impairment.

Despite this promise, the capacity of DMHIs to bridge treatment gaps remains limited [22]. The marketplace for DMHIs remains inefficient, with 90% use of MHAs overall being held by the 2 most popular MHAs available [23]. Moreover, although there have been growing efforts to increase the standards and rigor of the field [24], most studies have focused on feasibility and acceptability [25-27], with fewer than 5% empirically validated [28,29]. Even when DMHIs have been shown to be effective in rigorous trials, the potential to reproduce these results in real-world settings has been restricted by the overall lack of engagement and sharp attrition rates [20,30,31]. Overall, the clinical use of DMHIs has been disappointing given the low rates of uptake and engagement [32], with over 50% of the total DMHIs having little to no monthly engagement [33].

### Added Support for DMHIs

The most common solution to attrition and low engagement is to provide users with personalized feedback [19] and human support designed to personalize DMHIs through supportive text messages, phone calls, personalized feedback, monitoring, and troubleshooting [34-37]. One popular model of human support is supportive accountability [35], in which a supportive guide or coach, perceived as trustworthy, kind, and competent, provides encouragement and holds the user accountable for completing an intervention. This can increase motivation, takes less time than providing direct service, and can be done via both synchronous and asynchronous channels. There is growing evidence that human support of this nature can increase users’ engagement with technology-delivered interventions [34] as well as intervention outcomes [7,36]. For example, a meta-analysis of 66 unique experimental comparisons showed that when DMHI use was supplemented by synchronous or asynchronous support, the effects were double compared with unsupported DMHI use [7].

Despite these promising trends, research on the role of human support in DMHIs is relatively new, and important questions remain unresolved. The effectiveness of human support may vary across populations and the issues that DMHIs are designed to address. Some reviews of the effectiveness of coaching in DMHIs have focused on mood disorders including anxiety disorders and depression [38,39], whereas others have not specified diagnosis or symptom level as inclusion criteria. The effect of human support may also vary based on the support provider. Some studies rely on support by a clinician or therapist [40-42], whereas others deploy paraprofessional support providers such as research or clinical staff, technicians, or e-coaches [7,43-46]. Finally, results may vary on the basis of meta-analysis quality [47].

Altogether, these remaining questions have implications for whether and under what circumstances human support should be deployed. Given the heterogeneity of approaches and the potential costs and benefits of adding a coaching component to DMHI, a systematic review of the role of human support is required [39]. Whereas meta-analyses allow for quantitative comparisons exploring specific research questions, meta-reviews (systematic reviews of empirical meta-analyses on a given topic) can synthesize the findings across several meta-analyses to create a comprehensive depiction of the current state of the field and determine the empirical quality of the evidence from these meta-analyses. Given the inconsistencies in research testing human support for DMHI use, a meta-review can reveal study design variations and limitations, allowing researchers to evaluate such inconsistencies across the literature [48-50]. Although there have been some meta-reviews investigating the effects of DMHIs on mental health [8,51], only 1 scoping review has examined the role of human support in DMHI use [39]. The scoping review by Bernstein et al [39] included both quantitative and qualitative findings, was limited to cognitive behavioral approaches, and focused only on DMHIs delivered via MHAs. Of the 64 studies included, only 7 (11%) included quantitative comparisons of supported versus unsupported approaches. Of these, fewer than half (3/7, 43%) showed positive effects of human support, and the review reported mixed findings overall. The authors concluded that the field of support for DMHI use remains insufficient for drawing strong conclusions and highlighted the need for additional evaluations.

### This Study

A systematic meta-review of meta-analyses was conducted comparing human support with no support on DMHI outcomes. The goal of the review was to provide a more exhaustive representation of the effects of human support by summarizing the effects of human support on DMHIs (including MHAs and internet-based interventions) across various treatment outcomes, participant samples, and types of support providers and to evaluate the quality of evidence available.

## Methods

### Literature Search

A literature search was conducted to identify meta-analyses that investigated the use of human support for DMHIs on mental health outcomes. The search was restricted to meta-analyses available in English and included a comparison of mental health outcomes when human support was provided versus when no support was provided.

### Search Strategy

We searched the MEDLINE, PubMed, and PsycINFO electronic databases for relevant articles using key terms related to DMHIs, with filters for meta-analyses, availability in English (based on the primary researchers’ language fluency), and year of publication since 2011. The MEDLINE and PubMed searches were completed on August 30, 2021, and the PsycINFO search was completed on September 6, 2021. To complete the most comprehensive review, the reference list of an unpublished meta-analysis on technology-delivered interventions was also searched for relevant articles. The full search terms were as follows: (“digital,” OR “mHealth,” OR “eHealth,” OR “web-based,” OR “internet-based,” OR “mobile phone,” OR “smartphone,” OR “internet interventions,” OR “apps,” OR “artificial intelligence,,” OR “technology-delivered intervention” OR “mobile mental health intervention,” OR “digital mental health intervention,” OR “internet-delivered”) AND (“mental health,” OR “depression,” OR “depressive symptoms,” OR “depressive disorders,” OR “anxiety,” OR “affective symptoms,” OR “anxiety disorders,” OR “mood disorders,” OR “stress,” OR “PTSD,” OR “suicidal ideation,” OR “psychological distress”). The term “virtual” was intentionally not included in the search terms, despite its growing popularity in the literature since the start of the COVID-19 pandemic. However, our interpretation of the literature is that “virtual” seems to be used as a descriptor for how synchronous, person-delivered interventions are delivered via technology. In this paper, we focused on technology-delivered interventions in which the core mental health skills are delivered through the digital platform via reading, didactics, games, tasks, etc (instead of by another human on a digital platform).

### Exclusion and Inclusion Criteria

**Figure 1:**
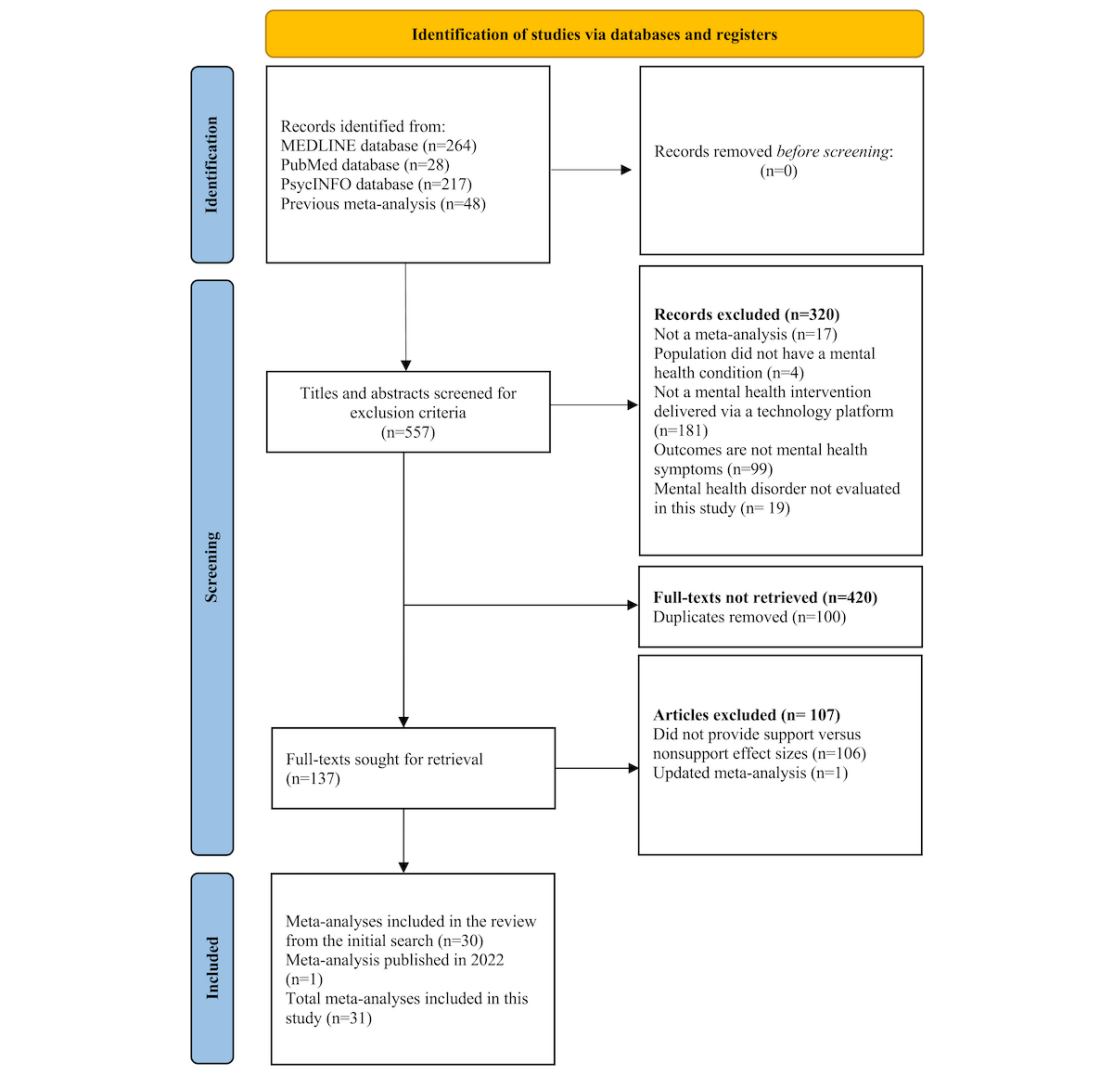
Selection of meta-analyses.

On the initial search, article abstracts were screened for inclusion criteria. Articles were included if they met the following criteria: (1) the study had a quantitative meta-analysis study design; (2) the intervention targeted mental health symptoms and was delivered via a technology platform (excluding person-delivered interventions mediated through telehealth, text messages, or social media); (3) the outcome variables included mental health symptoms such as anxiety, depression, stress, posttraumatic stress disorder (PTSD) symptoms, or a number of these symptoms together; and (4) the study included quantitative comparisons of outcomes in which human support versus those when no or minimal human support (excluding solely engagement reminders) was provided. Dissertations were included if they met the inclusion criteria. Two authors (SA and MJ) independently filtered and selected the meta-analyses based on the inclusion and exclusion criteria described earlier. Duplicates across search sources were removed, and the full texts of the remaining studies were screened for the inclusion criteria. One study that met the criteria was excluded in the last phase, as a more recently updated meta-analysis of the topic accounted for all the relevant primary studies. During manuscript preparation, the authors became aware of an additional meta-analysis that met the inclusion criteria [52]; this study was included in the final list of articles. See Figure 1 for the selection of meta-analyses.

### Coding, Data Extraction, and Synthesis Strategy

The final list of included meta-analyses was cross-checked by 3 authors (SA, MJ, and AE). The following data were extracted from each of the included meta-analyses: (1) authors and year of publication of the meta-analysis; (2) number of studies included in the quantitative synthesis (meta-analysis); (3) design of the studies included in the meta-analysis; (4) participant populations of studies included in the meta-analysis; (5) type of DMHI examined in the meta-analysis; (6) main mental health outcome variables examined in the meta-analysis; (7) the meta-analysis’s definition of support and nonsupport; (8) effect sizes and CIs for all levels of support quantitatively conducted in the meta-analysis for mental health outcomes; and (9) *P* values for the difference between levels of support for mental health outcomes. Data extraction of the included studies was conducted in duplicate by the same 3 authors. Disagreements or questions were resolved between the 3 authors when needed, with questions and concerns being brought to the corresponding author (JR). To increase the integrity of our study, the PRISMA (Preferred Reporting Items for Systematic Reviews and Meta-Analyses) [53] guidelines were followed. As the included studies were meta-analyses, risk of bias was not assessed. All authors contributed to the comparisons of effect sizes by outcome domain to examine the efficacy of human-supported DMHIs; no quantitative analyses were conducted in this meta-review.

### Quality Assessment

Quality assessment was conducted for each meta-analysis using the Assessment of Multiple Systematic Reviews (AMSTAR) 2, as provided in the study by Shea et al [54]. AMSTAR allows researchers to rate the quality of systematic reviews and meta-analyses by indicating if the authors of the article completed a specific set of tasks, such as performing study selection in duplicate and describing the characteristics of the included studies in sufficient detail. Three authors (SA, MJ, and AE) evaluated the quality of the included studies jointly until a 95% agreement was met, after which the remaining studies were independently evaluated. Items with No, Partial Yes, and Yes options were given scores of 0, 1, and 2, respectively, and items with No and Yes options were given scores of 0 and 2, respectively. A percentage score was calculated for each study. For this meta-review, meta-analyses that satisfied at least 70% of the eligible AMSTAR 2 items were considered higher quality meta-analyses, whereas those with 50% to 69% completion were considered medium quality, and those with less than 50% were considered low quality. This method was informed by a previous meta-review [55].

### Overlap Analysis

An overlap analysis was conducted to determine the percentage of primary studies evaluated across all meta-analyses that overlapped. Three authors (SA, MJ, and AE) independently extracted the reference citations of all primary studies included in each meta-analysis. The corrected covered area (CCA) [56] was estimated to determine the degree of overlap of the primary studies in the included meta-analyses. The CCA was calculated using a script that applies the calculation formula provided by the authors using the citation matrix [56]. Following these guidelines, CCA scores from 0% to 5% were considered to have slight overlap, those from 6% to 10% were considered to have moderate overlap, those from 11% to 15% were considered to have high overlap, and those greater than 15% were considered to have very high overlap. The results of the calculation showed a CCA value of 0.030 (3%), indicating slight overlap across meta-analyses. We suspect that the heterogeneity of the topics of the meta-analyses led to primary studies not meeting the inclusion criteria for multiple meta-analyses.

## Results

### Description of Included Studies

The initial search identified 557 studies, from which 420 were excluded. Most (n=320) were excluded by screening the title and abstract, whereas others (n=100) were duplicates. The full texts of the remaining 137 studies were obtained and reviewed, and 107 further studies were excluded. After reviewing 505 unique primary studies, 31 meta-analyses on the effectiveness of DMHIs on mental health outcomes comparing human support versus no support were included in this meta-review. The PRISMA flowchart for the inclusion of studies in the meta-review is shown in Figure 1. Characteristics (number of studies, study design, participant population, DMHI type, outcomes, and quality assessment) of the included meta-analyses are shown in Table 1. See Multimedia Appendix 1 [3,34,47,54] for a list of studies that initially appeared to meet the inclusion criteria but were later excluded. Given that the focus of this meta-review was to examine the effects of human support, Table 2 includes the definitions of human support by study. The meta-analyses varied considerably in terms of the amount of detail provided. Some studies included varying levels of human support (eg, full support vs some support vs no support), whereas others only compared supported with unsupported cases.

**Table 1:** Meta-analyses included for final analyses.

Study	Number of studies	Study design	Participant population	DMHI^a^ type	Outcome variables	Quality assessment
Carolan et al [57], 2017	21	RCTs^b^	Employed participants aged ≥18 years; targeted populations (psychological) and universal population	Occupational digital mental health interventions	Multiple problems (stress, depression, and psychological distress)	Low
Cheng et al [47], 2020	14	RCTs	People with HIV or AIDS and with clinical or subclinical depression	Technology-delivered psychotherapeutic interventions	Depression	Low
Conley et al [58], 2016	48	Mixed (reports, RCT, or quasi-experimental control design)	Higher education students; universal prevention or indicated prevention	Technological mental health prevention programs	Mental health–related outcomes	Low
Cowpertwait and Clarke [59], 2013	18	RCTs	Depressed adults	Web-based psychological interventions	Depression	Low
Domhardt et al [4], 2019	34	RCTs	Adults with specific phobia, social anxiety disorder, panic disorder, agoraphobia, or generalized anxiety disorder at baseline	Internet- and mobile-based interventions for anxiety	Anxiety	Medium
Firth et al [60], 2017	18	RCTs	No restrictions based on diagnosis or any clinical or demographic traits	Smartphone- delivered mental health interventions	Depression	Low
Fu et al [61], 2020	22	RCTs	Individuals with mental health problems in low-income and middle-income countries	Digital psychological interventions	Mental health issues	Medium
Grist et al [62], 2019	34	RCTs	Youth with anxiety or depression	Technology-delivered interventions for depression and anxiety	Multiple problems (anxiety or depression symptoms)	Low
Harrer et al [63], 2018	48	RCTs	University students	Internet-delivered psychological interventions	Depression and anxiety	Medium
Heber et al [5], 2017	23	RCTs	Adult participants who experienced stress	Web- and computer-based stress management interventions	Multiple problems (stress, depression, and anxiety)	Medium
Kampmann et al [64], 2016	37	RCTs	Adults who meet diagnostic criteria for social anxiety disorder	iCBT^c^	Anxiety (social anxiety)	Medium
Kuester et al [65], 2016	20	RCTs	Adults with clinical or subclinical PTSD^d^	Internet-based interventions. CBT^e^ and expressive writing. Guided vs unguided comparisons only done with internet-based CBT	PTSD	Low
Leung et al [52], 2022	13	RCTs	Participants aged 16-64 years, clinical or subthreshold mental health symptoms	Digital intervention targeting mental health	Multiple mental health problems	Medium
Li et al [66], 2014	8	RCTs	No limitations on the participants’ age or the significance of the depression symptoms	Game-based digital interventions for depression	Depression	Medium
Linardon et al [7], 2019	66	RCTs	All ages	App-supported smartphone interventions for mental health problems	Depression and anxiety (generalized anxiety)	Low
Mehta et al [46], 2019	25	RCTs	People with chronic health conditions	iCBT	Depression and anxiety	Low
Pang et al [41], 2021	18	RCTs	Adults with depression that was diagnosed by a physician or by any well-validated depression scales	Web-based self-management interventions for depression	Depression	Medium
Păsărelu et al [38], 2017	19	RCTs	Adult participants (aged ≥18 years) with either symptoms or a primary diagnosis of anxiety or unipolar depression	Transdiagnostic or tailored interventions, based on a CBT protocol; delivered on the web, via the internet (both self-help and clinician-delivered)	Anxiety	Low
Phillips et al [44], 2019	34	RCTs	Adults with any mental health condition in an employee population for any occupation	Occupational e–mental health interventions (information and communication technology based)	Stress, depression, and anxiety	Medium
Richards and Richardson [40], 2012	23	RCTs	Adults with depression (self-report or diagnosis), established using valid and reliable measures, who may also have had comorbidity	Computer-based psychological treatments for depression	Depression	Low
Sherifali et al [45], 2018	13	Mixed (RCTs or controlled clinical trials)	Informal caregivers aged ≥18 years who were currently providing caregiving support to adults aged ≥18 years living in the community with at least 1 chronic condition	Internet-based interventions	Depression and anxiety	Medium
Sijbrandij et al [67], 2016	12	RCTs	Individuals with subclinical or clinical PTSD	iCBT for PTSD	PTSD and depression	Low
Simmonds-Buckley et al [68], 2020	24	RCTs	Adults aged ≥18 years with depression or anxiety	Web-based or smartphone app intervention	Depression and multiple problems (anxiety and Stress)	Medium
Spijkerman et al [69], 2016	15	RCTs	Adults aged ≥18 years	Web-based MBIs^f^	Stress, depression, and anxiety	Low
Stratton et al [70], 2017	23	RCTs and pre- or posttrials	Current paid employment and working age adults	eHealth-based intervention	Multiple problems (depression, anxiety, and stress outcomes)	Low
Sztein et al [6], 2018	14	RCTs	Adults aged ≥18 years with depression	iCBT	Depression	Medium
Thompson et al [42], 2021	25	RCTs	Any adult population (aged ≥18 years); see the article for participant details	Internet-based acceptance and commitment therapy	Depression and anxiety	High
Twomey et al [71], 2020	12	RCTs	Adults with elevated depressive symptoms	Individually tailored computer-assisted CBT program for depression	Depression	Medium
Versluis et al [72], 2016	27	RCT, pre- or postdesign	Clinical and healthy samples	Ecological momentary interventions	Multiple problems (anxiety, depression, and perceived stress)	Low
Victorson et al [73], 2020	43	RCTs	Clinical and healthy nonclinical populations. The average participant age was 40.5 years	Technology-enabled mindfulness-based programs	Anxiety, depression, and stress	Low
Wright et al [43], 2019	40	RCTs	Participants with depression (clinically diagnosed or diagnosed by standardized assessments)	Computer-assisted CBT	Depression	Low

^a^DMHI: digital mental health intervention.

^b^RCT: randomized controlled trial.

^c^iCBT: internet- delivered cognitive behavioral therapy.

^d^PTSD: posttraumatic stress disorder.

^e^CBT: cognitive behavioral therapy.

^f^MBI: mindfulness-based intervention.

**Table 2:** Description of human support by meta-analysis.

Study	Description of human support	Description of no human support
Carolan et al [57], 2017	Studies varied in who provided support: 70% of the studies described the support as coming from a therapist or coach, 20% had a coordinator or member of staff providing support, and 10% described support as a clinical psychologist	Self-guided DMHI^a^
Cheng et al [47], 2020	Professional support	Self-guided DMHI
Conley et al [58], 2016	Participants received prompts, reminders, feedback, or guidance through emails, and some personal monitoring of the intervention	Self-administered DMHIs, in which assistance was provided only for assessment purposes or to offer a brief introduction to the technology
Cowpertwait and Clarke [59], 2013	Human-supported	Self-guided DMHI
Domhardt et al [4], 2019	Continuous therapeutic support	Self-help DMHI, with therapist contact for assessment (if at all)
Firth et al [60], 2017	Involved “in-person” (ie, human) feedback	No in-person feedback
Fu et al [61], 2020	Presence of guidance	Absence of guidance
Grist et al [62], 2019	Supported: minimal contact therapy (“active involvement of therapist, help in applying specific therapeutic techniques, >90 min of time”); some support: predominantly unguided defined as predominantly self-administered (“giving initial therapeutic rationale, direction on how to use the program and periodic check-ins, <90 min of time”)	Purely unguided defined as purely self-administered (“therapist contact for assessment at most”)
Harrer et al [63], 2018	Individual feedback	Unguided DMHI
Heber et al [5], 2017	Guided with regular written feedback	Unguided with no support or only technical support
Kampmann et al [64], 2016	Guided internet-delivered cognitive behavioral therapy	Unguided internet-delivered cognitive behavioral therapy
Kuester et al [65], 2016	Therapeutic support from a therapist (“in remote contact with the client and provided therapeutic feedback messages”)	No therapeutic support (“programs that were either fully automated, provided only nontherapeutic moderation such as the supervision of forum posts or solely technical assistance”)
Leung et al [52], 2022	Nonclinician (eg, peers, research assistants, or other lay persons) or clinician (ie, psychiatrists, psychologists, therapists, social workers, graduate students in a mental health–related field, or students completing clinical practicum training)	Unguided
Li et al [66], 2014	Therapist involved (minimal contact therapy and therapy administrated)	No therapist involved (self-administered and predominately self-help)
Linardon et al [7], 2019	Studies that offered professional guidance (eg, regular supportive text messages, phone calls, or personalized feedback from therapists or research staff)	Studies that did not offer professional guidance
Mehta et al [46], 2019	Therapist-guided (“usually involve weekly contact with a web-based therapist or guide, either through asynchronous web-based messaging or by telephone”)	Self-guided DMHI (“participants do not have regular contact with a therapist”)
Pang et al [41], 2021	Therapist guidance group (“group communicating with the therapist”); virtual health indicator guidance group (“group communicating with the virtual health care provider”)	No therapist guidance group (“group not communicating with the therapist”)
Păsărelu et al [38], 2017	Experienced clinical psychologists and supervised students	Self-guided DMHI
Phillips et al [44], 2019	Studies with guidance provided different types of human support (eg, regular calls by a clinical study officer, feedback from a clinical psychologist on home assignments, regular guidance from trained e-coaches, peer group discussions, and virtual class meetings)	Without guidance
Richards and Richardson [40], 2012	Therapist-supported studies included a clinician who offered postsession feedback and support or a clinician-delivered intervention	Completely self-administered
Sherifali et al [45], 2018	Internet-based information or education plus professional psychosocial support	Internet-based information or education only
Sijbrandij et al [67], 2016	Therapist-assisted (email, telephone calls, in-person support)	Self-help
Simmonds-Buckley et al [68], 2020	Predominantly therapist delivered	Self-administered DMHI
Spijkerman et al [69], 2016	Therapist guidance	Without therapist guidance
Stratton et al [70], 2017	Feedback provided, rather than just technical support	Self-help
Sztein et al [6], 2018	Clinician was in some way involved in the dissemination of information to the study participants, whether through discussion forums, email, telephone, etc	Self-guided
Thompson et al [42], 2021	Therapist-guided (included phone calls, personalized written messages and feedback, tailored emails, face-to-face meetings, and automated text messages or emails)	Not guided (although may have included automated text messages or emails)
Twomey et al [71], 2020	Clinician or technician guidance	Without guidance
Versluis et al [72], 2016	DMHI was included in a “treatment package” and was supported by a mental health professional; the “treatment package” could include the DMHI and therapy *or* DMHI and continued feedback (on homework assignments or messages to improve adherence)	Stand-alone DMHI
Victorson et al [73], 2020	Human-supported	Without human support
Wright et al [43], 2019	Clinician assisted or technician assisted	Unsupported DMHI

^a^DMHI: digital mental health intervention.

### Methodological Quality of the Meta-analyses

On average, the meta-analyses achieved 49% completion of all AMSTAR 2 items, ranging from 25% to 81% satisfaction. A total of 17 studies were rated as low quality, 14 as medium quality, and 1 as high quality based on the AMSTAR 2 quality assessment scale. See Multimedia Appendix 2 [4-7,38,40-45,47,57-73] for a list of percentages achieved for each meta-analysis included.

### Summary of All Effect Sizes

Of the 45 effect sizes reported, almost half (n=22, 48%) showed that human-supported interventions were significantly more effective than unsupported interventions. Only 9% (4/45) of effect sizes showed unsupported as significantly more effective (Table 3). A total of 28% (13/45) of effect sizes showed a potential trend toward supported interventions; however, these results were not significant.

**Table 3:** Effect sizes from included studies by outcome domain.

Meta-analysis			Effect size type		Human-supported					Not supported					Significant difference		
					Effect size		CI		Effect size			CI		Yes or no			Direction
**Anxiety**																	
	Victorson et al [73], 2020	Effect size differences^a^		Mean −0.07 (SD 0.88)		—^b^		Mean −0.14 (SD 0.47)			—		No			No difference; not significant	
	Sherifali et al [45], 2018	SMD^c^		−0.36		−0.66 to −0.07		−0.42			−0.65 to −0.19		Yes			Unsupported significantly; more effective	
	Phillips et al [44], 2019	Hedges *g*		0.48		0.16 to 0.80		0.26			0.10 to 0.41		Yes			Guided significantly; more effective	
	Thompson et al [42], 2021	Hedges *g*		0.28		0.18 to 0.38		0.16			−0.10 to 0.42		No			Guided slightly more effective; not significant	
	Kampmann et al [64], 2016	Hedges *g*		0.87		0.72 to 1.02		0.78			0.50 to 1.05		No			Guided slightly more effective; not significant	
	Kampmann et al [64], 2016	Hedges *g*		0.47		0.15 to 0.78		0.19			−0.08 to 0.46		No			Guided slightly more effective; not significant	
	Linardon et al [7], 2019	Hedges *g*		0.53		0.36 to 0.70		0.21			0.12 to 0.30		Yes			Guided significantly; more effective	
	Spijkerman et al [69], 2016	Hedges *g*		0.26		0.02 to 0.50		0.19			−0.06 to 0.43		No			Guided slightly more effective; not significant	
	Harrer et al [63], 2018	Hedges *g*		0.27		0.02 to 0.52		0.25			0.02 to 0.49		No			Guided slightly more effective; not significant	
	Domhardt et al [4], 2019	SMD		−0.39		−0.59 to −0.18		—			—		Yes			Guided significantly; more effective	
	Păsărelu et al [38], 2017	Hedges *g*		0.87 (clinical psychologist); 0.76 (supervised students)		0.48 to 1.26; 0.56 to 0.96		0.54			—		Yes			Guided significantly; more effective	
	Mehta et al [46], 2019	Cohen *d*		0.54 (SE 0.08)		—		Mean 0.57 (SE 0.12)			—		No			Unsupported slightly more effective; not significant	
**Depression**																	
	Victorson et al [73], 2020	Effect size differences		Mean −0.12 (SD 0.93)		—		Mean−0.46 (SD 0.79)			—		No			No difference; not significant	
	Richards and Richardson [40], 2012	Cohen *d*		0.78		−0.92 to −0.64		0.36			−0.61 to −0.10		Yes			Guided significantly; more effective	
	Sherifali et al [45], 2018	SMD		−0.34		−0.63 to −0.05		−0.31			−0.50 to −0.11		Yes			Guided significantly; more effective	
	Phillips et al [44], 2019	Hedges *g*		0.48		0.33 to 0.63		0.23			0.08 to 0.37		Yes			Guided significantly; more effective	
	Thompson et al [42], 2021	Hedges *g*		0.45		0.34 to 0.56		0.14			−0.022 to 0.29		Yes			Guided significantly; more effective	
	Firth et al [60], 2017	Hedges *g*		0.137		−0.08 to 0.35		0.465			0.30 to 0.63		Yes			Unsupported significantly; more effective	
	Li et al [66], 2014	Cohen *d*		−0.44		−0.73 to −0.15		−0.54			−0.86 to −0.21		Yes			Unsupported significantly; more effective	
	Linardon et al [7], 2019	Hedges *g*		0.48		0.34 to 0.62		0.23			0.15 to 0.31		Yes			Guided significantly; more effective	
	Cheng et al [47], 2020	Cohen *d*		0.22		—		0.27			—		No			Unsupported slightly more effective; not significant	
	Cowpertwait and Clarke [59], 2013	Hedges *g*		0.48		0.39 to 0.57		0.32			0.23 to 0.41		Yes			Guided significantly; more effective	
	Spijkerman et al [69], 2016	Hedges *g*		0.29		0.06 to 0.53		0.29			0.03 to 0.55		No			No difference, not significant	
	Sijbrandij et al [67], 2016	Hedges *g*		0.66		0.36 to 0.96		0.55			0.12 to 0.98		No			Guided slightly more effective; not significant	
	Harrer et al [63], 2018	Hedges *g*		0.28		−0.02 to 0.57		0.15			0.06 to 0.25		No			Guided slightly more effective; not significant	
	Simmonds-Buckley et al [68], 2020	Hedges *g*		0.61 (predominantly therapist delivered); 0.39 (minimal contact)		—; 0.16 to 0.62		0.30			0.15 to 0.45		No			Guided slightly more effective; not significant	
	Mehta et al [46], 2019	Cohen *d*		0.64 (SE 0.15)		—		Mean 0.45 (SE 0.18)			—		Yes			Guided significantly; more effective	
	Twomey et al [71], 2020	Hedges *g*		0.57		0.36 to 0.78		0.47			0.32 to 0.62		No			Guided slightly more effective; not significant	
	Wright et al [43], 2019	Hedges *g*		0.673		0.546 to 0.801		0.239			0.115 to 0.364		Yes			Guided significantly; more effective	
	Pang et al [41], 2021	Hedges *g*		−0.60 (therapist); −0.27 (web-based health care provider)		−0.81 to −0.38; −0.58 to 0.05		−0.17			−0.40 to 0.06		Yes			Guided significantly; more effective	
	Sztein et al [6], 2018	SMD		0.73		0.58 to 0.87		0.79			0.55 to 1.03		No			No statistically significant difference	
**PTSD^d^**																	
	Kuester et al [65], 2016	Hedges *g*		0.8		0.62 to 0.98		0.54			0.22 to 0.86		No			Guided slightly more effective; not significant	
	Sijbrandij et al [67], 2016	Hedges *g*		0.89		0.70 to 1.08		0.5			0.22 to 0.78		Yes			Guided significantly; more effective	
**Stress**																	
	Spijkerman et al [69], 2016	Hedges *g*		0.89		0.65 to 1.12		0.19			−0.01 to 0.38		Yes			Guided significantly; more effective	
	Victorson et al [73], 2020	Effect size differences		Mean −0.20 (SD 0.49)		—		Mean −1.63 (SD 1.8)			—		Yes			Unsupported significantly; more effective	
	Phillips et al [44], 2019	Hedges *g*		0.76		0.44 to 1.08		0.38			0.19 to 0.56		Yes			Guided significantly; more effective	
	Conley et al [58], 2016	Hedges *g*		0.55		0.37 to 0.72		0.28			0.14 to 0.40		Yes			Guided significantly; more effective	
	Heber et al [5], 2017	Cohen *d*		0.64		(0.50 to 0.79)		0.33			(0.20 to 0.46)		Yes			Guided significantly; more effective	
	Grist et al [62], 2019	Hedges *g*		0.87 (minimal contact therapy);0.81 (predominantly self-help)		0.68 to 1.06; −0.68 to −2.31		0.24			0.10 to 0.38		Yes			Guided significantly; more effective	
	Simmonds-Buckley et al [68], 2020	Hedges *g*		0.60 (minimal therapist contact); 0.47 (predominantly self-help)		0.36 to 0.83; 0.11 to 0.83)		0.23			0.09 to 0.36		Yes			Guided significantly; more effective	
	Carolan et al [57], 2017	Hedges *g*		0.39		0.18 to 0.61		0.34			0.16 to 0.53		No			Guided slightly more effective; not significant	
	Stratton et al [70], 2017	Hedges *g*		0.27		—		0.22			—		No			Guided slightly more effective; not significant	
	Versluis et al [72], 2016	Hedges *g*		0.73 (mental health provider); 0.38 (DMHI + care as usual)		0.57 to 0.88; 0.11 to 0.64		0.45			0.22 to 0.69		Yes			Guided significantly; more effective	
	Fu et al [61], 2020	Hedges *g*		0.61		0.43 to 0.78		0.6			0.35 to 0.86		No			Guided slightly more effective; not significant	
	Leung et al^e^ [52], 2022	Hedges *g*		−0.17		−0.23 to 0.11		—			—		Yes			Guided significantly; more effective	

^a^Victorson et al [73] reported differences in effect sizes for supported versus unsupported interventions.

^b^Not available.

^c^SMD: standardized mean difference.

^d^PTSD: posttraumatic stress disorder.

^e^Posttreatment SMD effect size overall comparison.

#### Outcome Domains

See Table 4 for the number of effect sizes showing effects in favor of supported interventions, the number of effect sizes showing effects in favor of unsupported interventions, and the number of effect sizes showing no significant differences between supported and unsupported interventions based on the characteristics of the studies. No patterns emerged regarding the effects of human support across outcome domains.

In particular, 26% (12/45) of effect sizes represented anxiety symptoms. Of these, 4 suggested that supported DMHIs resulted in significantly lower anxiety symptoms compared with unsupported DMHIs [4,7,38,44]. Only 1 meta-analysis found that unsupported interventions had significantly higher effects [45]. Among the meta-analyses, 5 effect sizes used clinical samples (with diagnosed or elevated clinical symptoms). Three of those indicated significant effects for human-supported DMHIs, and 2 effect sizes revealed null results comparing supported and unsupported interventions. When examining the studies that used clinical samples, those that found supported DMHIs more effective than unsupported DMHIs included individuals with “any mental health condition” [44], anxiety disorders [4], and anxiety or unipolar depression [38]. Interestingly, the study that examined DMHIs for individuals with social anxiety found no significant differences based on supported or unsupported DMHIs using 2 effect sizes [64].

A total of 19 meta-analyses examined the effect sizes of DMHIs on depression symptoms. Nine of those meta-analyses suggested that supported DMHIs result in significantly lower depression symptoms compared with unsupported DMHIs [7,40-46,59]. Two meta-analyses found that unsupported DMHIs were significantly more effective than supported DMHIs [60,66]. When focusing exclusively on studies of individuals with elevated symptoms of depression [6,40,41,43,71], supported DMHIs were more effective in reducing depressive symptoms than unsupported DMHIs (with 3 studies showing significant findings and 2 failing to find significant differences).

Two meta-analyses measured the effect sizes of DMHIs for the treatment of PTSD symptoms. One meta-analysis suggested that supported DMHIs result in significantly lower PTSD symptoms compared with unsupported DMHIs [67]. The other meta-analysis did not find statistically significant effects of human support [65]. Both studies included samples of individuals with elevated PTSD symptoms.

Three meta-analyses examined the effect sizes of DMHIs on stress. Two suggested that supported DMHIs result in significantly lower stress compared with unsupported DMHIs [44,69], whereas 1 found the opposite effect [73]. Only 1 meta-analysis [44] included a clinically elevated sample, which focused on individuals with “any mental health condition.” The other 2 studies included unselected samples of participants.

Finally, 9 meta-analyses examined the effect sizes of DMHIs on multiple mental health problems. Six of those meta-analyses suggested that supported DMHIs result in significantly lower mental health symptoms compared with unsupported DMHIs [5,52,58,62,68,72]. No meta-analyses found stronger effects of unsupported DMHIs for multiple mental health symptoms.

**Table 4:** Number of effect sizes showing effects (N=45).

				Total number of effect sizes reported		Human-supported interventions had significantly greater effects, n (%)		No significant differences between human-supported and unsupported interventions, n (%)		Unsupported interventions had significantly greater effects, n (%)
**Outcome domains**										
	Anxiety		12		4 (33)		7 (58)		1 (8)	
	Depression		19		9 (47)		8 (42)		2 (10)	
	PTSD^a^		2		1 (50)		1 (50)		0 (0)	
	Stress		3		2 (66)		0 (0)		1 (33)	
	Multiple		9		6 (66)		3 (33)		0 (0)	
**Sample characteristics**										
	**Clinical or subclinical**		21		12 (57)		9 (42)		0 (0)	
		Anxiety disorders	1		1 (100)		0 (0)		0 (0)	
		Social anxiety	2		0 (0)		2 (100)		0 (0)	
		Depression	6		3 (50)		3 (50)		0 (0)	
		Anxiety disorders or depression	4		3 (75)		1 (25)		0 (0)	
		PTSD	3		1 (33)		2 (66)		0 (0)	
		Unrestricted mental health conditions	5		4 (80)		1 (20)		0 (0)	
	Unrestricted samples		24		10 (41)		10 (41)		4 (16)	
**Quality of RCT^b^**										
	High		2		1 (50)		1 (50)		0 (0)	
	Medium		19		9 (47)		8 (42)		2 (10)	
	Low		24		12 (50)		10 (41)		2 (8)	
**Type of human support**										
	Clinically trained^c^		17		8 (47)		8 (47)		1 (5)	
	Mixed^d^		9		7 (77)		2 (22)		0 (0)	
	Unclear^e^		19		7 (36)		9 (47)		3 (15)	

^a^PTSD: posttraumatic stress disorder.

^b^RCT: randomized controlled trial.

^c^Clinically trained is defined as a therapist, clinical psychologist, or clinical psychology trainee.

^d^Mixed support providers included both clinically trained individuals and individuals who did not have clinical training providing support for DMHIs.

^e^Unclear means that the authors did not provide information about the type of support provider in the meta-analysis.

#### Sample Characteristics

When examining effect sizes in randomized controlled trials (RCTs) that included participants with clinical or subclinical levels of symptoms, there were more significant effect sizes showing that human support increases intervention efficacy compared with no significant differences based on support. When examining unrestricted samples, the results were more mixed (Table 4).

Clinical or subclinical samples were further broken down by condition. One effect size reported that human-supported DMHIs were more effective than unsupported DMHIs for individuals with a variety of anxiety disorders, whereas 2 effect sizes found no significant effects of human support when the samples only included individuals with social anxiety specifically. Six effect sizes were reported for samples with depression; effect sizes were split between those favoring supported DMHIs and those that did not find significant differences between supported and unsupported DMHIs. Four effect sizes were reported for samples with anxiety or depression; most demonstrated that human-supported DMHIs were more effective than unsupported DMHIs. The results suggested a different pattern for individuals with PTSD, with most (2 out of 3) effect sizes suggesting no significant differences between supported and unsupported DMHIs. Finally, 4 of 5 effect sizes suggested that human-supported DMHIs were more effective than unsupported DMHIs among samples with mixed mental health conditions.

#### Quality of RCTs

Across high-, medium-, and low-quality RCTs, the percentage of effect sizes showing positive effects versus no effects was similar (Table 4). When only considering those studies with high- or medium-quality AMSTAR 2 ratings, the results seem to be split between effect sizes in favor of human-supported DMHIs and those revealing no significant differences between supported and unsupported DMHIs.

#### Support Provider Characteristics

Four of the studies included in this meta-review examined whether the supportive person’s training was related to DMHI effectiveness [4,38,40,52]. Three studies found no significant differences in effect sizes between experienced individuals providing support (eg, licensed clinicians) and individuals with less experience (eg, students or nonclinicians) [4,38,52]. One study [40] found significant differences between therapist-supported DMHIs and administrative staff members providing support for DMHIs, with therapist-supported DMHIs yielding higher effect sizes.

Of those meta-analyses that only included individuals with clinical training (eg, therapists, clinical psychologists, and clinical psychology trainees), approximately half of the effect sizes were in support of human support and approximately half found no significant differences between supported and nonsupported DMHIs; 1 meta-analysis found that the unsupported DMHIs were more effective. In contrast, among studies that reported that the included primary studies included a mix of supportive individuals (both clinically trained individuals and individuals without clinical training), effect sizes were more likely to be in favor of human support. Of those meta-analyses that did not define the type of support providers, the effect sizes were mixed in terms of efficacy of human-supported DMHIs (Table 4).

## Discussion

### Principal Findings

A systematic meta-review of meta-analyses was conducted that compared the effects of human support or DMHIs with no support on mental health symptoms. The effects of human support on treatment outcomes, participant samples, and types of support providers were examined. Results from 31 meta-analyses representing 505 unique primary studies have been reported. Almost half (22/45, 48%) of the effect sizes revealed that supported interventions had significantly stronger effects compared with unsupported interventions. Only 9% (4/45) of effect sizes described the significantly stronger effects of unsupported interventions. No clear pattern of results emerged in the outcome domain. Evidence for human-supported DMHIs was split for depression and PTSD symptoms; for anxiety symptoms, evidence suggested that there were largely no significant differences between human-supported and unsupported DMHIs. However, when multiple outcomes were assessed, human support for DMHIs appeared to be more effective than no support. Given the variable and number of studies across several outcomes and discrepant results, it would be premature to draw firm conclusions regarding the relative importance of human support for DMHIs across different outcome domains. Similarly, no clear pattern of results emerged for sample characteristics, with effect sizes largely split across those that did vs did not show the efficacy of added human support. The same was true regarding the quality of the meta-analysis.

Moreover, we did not find a clear pattern of results when comparing highly trained support providers (eg, clinicians) with paraprofessional-level support, suggesting that DMHIs do not need to be supported by individuals with extensive mental health training. This is promising for models of increasing access to mental health services and has implications for task-shifting mental health care as well as for therapeutic mentoring [74]. Unfortunately, 19 of the 45 effect sizes were from meta-analyses that did not define the training or background of the individuals providing support, greatly limiting our ability to draw strong conclusions about the role of background and training of support providers on the efficacy of human support. Although no clear patterns emerged in the outcome domain, sample characteristics, or provider background, we highlight a few promising trends that can guide future research and practice. Among DMHIs that target individuals with elevated mental health symptoms and specific mental health symptoms (depression, anxiety, and PTSD), human support appears to lead to stronger effects when compared with unsupported DMHIs. Future studies should explore this association.

Among the meta-analyses that included unrestricted samples (eg, open to adults), the results for human support were more mixed. Our review suggests that human support may play an important role in helping individuals with specific challenges engage with DMHIs that may be the most effective. Future research will need to further specify the conditions under which human support is most effective, disentangling the mechanisms through which it has its effects. Support may provide the structure and incentives to help individuals engage with DMHIs such that they are more effective. In addition, support may also provide a quasi-therapeutic alliance that increases motivation. Along these lines, Mohr et al [35] set forth a range of testable hypotheses pertaining to client motivation, alliance, and communications media, each of which should be more explicitly defined, tested, and manualized. Similarly, there is a need to specify the type of human support provided, as it can vary and may include postsession feedback, regular calls, feedback on assignments, regular supportive text messages, asynchronous web-based messaging, personalized feedback, tailored emails, or even face-to-face meetings [75].

### Limitations

Several limitations should be considered when interpreting these findings. First, our study was limited by the available meta-analytic study literature. This meta-review may have excluded primary studies that examined the effects of DMHIs and compared the effects of supported versus unsupported interventions but were not included in the meta-analyses. Second, our search was limited to studies published in English and may have excluded some otherwise meeting the inclusion criteria. Third, meta--reviews are constrained by the limitations in primary studies that have been summarized in the included meta-analyses, meaning that the original limitations of the primary research are not considered as the main findings are summarized. Furthermore, our meta-review used a thematic synthesis of the findings from the included meta-analyses, which could be vulnerable to issues of subjective interpretation [76]. In addition, this meta-review does not specify for whom human-supported DMHIs are most effective, as there are insufficient meta-analyses to draw firm conclusions by samples or diagnoses. Future research is necessary to investigate how background characteristics (eg, demographics and symptom severity) interact with the types of human support. It will also be important to continue to explore interactions between DMHI approaches (ie, MHAs and internet browser–based intervention) and human support, as different types of DMHIs may need extra support.

This meta-review was limited by the variable quality of evidence from the included meta-analyses. Of note, only 1 meta-analysis included in this review achieved a high-quality rating on the AMSTAR 2 guide, and most meta-analyses were rated as low quality. Overall, these ratings indicate that the meta-analyses included in this study demonstrated weakness in the core domains of experimental research methodology, hindering our confidence in drawing strong conclusions [54]. Moreover, the insufficient reporting of specific intervention characteristics in the included meta-analyses prevents us from providing discrete recommendations for future intervention protocols. One of the most important omissions from many meta-analyses was the description of individuals providing support. It should be noted that 19 of the 45 effect sizes that were reported in the meta-analyses did not provide this information with sufficient clarity for coding. Thus, our lack of specificity in this review reflects the current state of intervention reporting in the field. Without such details, it will be difficult to advance and improve the specificity with which human support procedures can improve DMHI outcomes.

### Comparison With Prior Work

To our knowledge, the review by Bernstein et al [39] is the only prior scoping review examining the role of human support in DMHI use. This study was able to expand on the work by Bernstein et al [39] by reviewing a more exhaustive set of DMHIs (rather than just MHAs with cognitive behavioral approaches) while focusing on higher-order quantitative comparisons of human support levels for DMHI use. However, similar to Bernstein et al [39], this meta-review was unable to draw strong conclusions based on the outcome of interest. Across studies in our meta-review, there did not appear to be a strong pattern of results when examining effect sizes across mental health outcomes (anxiety, depression, PTSD, stress, or multiple outcomes), sample characteristics, or meta-analysis quality. Understanding how the type of support may interact with diagnoses or challenges is critical. Our review found that supported DMHIs targeting anxiety symptoms among individuals with clinically elevated symptoms may be more effective than unsupported DMHIs targeting similar outcomes. However, we noted an important caveat in the results. For example, the findings did not hold for the study that examined meta-analyses focusing on individuals with social anxiety disorder [64]. Recent data from a human-supported, web-based anxiety program suggested that human support may negatively interact with social anxiety symptoms; some individuals enrolled in a web-based anxiety intervention reported that they did not want to talk on the phone with an intervention coach, citing their anxiety about speaking to strangers [76]. Some individuals cited this as a reason for dropping out of the intervention altogether. Given the mixed results in our study, we recommend that future research more closely examine how specific mental health challenges and interventions may benefit from (or be hindered by) specific models of human support.

This similarity notwithstanding, our meta-review provides insights for leveraging DMHIs. Bernstein et al [39] suggested their overall results of human support on DMHI outcomes to be mainly ambiguous. However, our study found that nearly half of the meta-analyses within this sample indicated human-supported interventions to have significantly better outcomes and that among those samples with clinical elevations, effects may be stronger. Less than 10% of the effect sizes showed stronger effects of unsupported DMHIs.

Although additional research is needed, our results highlight the important role that human support plays across various types of interventions, suggesting promise for reducing the global burden of mental health challenges and the lack of access to adequate care. They also suggest that positive effects of human-supported DMHIs are not limited to clinically severe cases, which offers promise for considering how these interventions may be useful in prevention settings as well.

### Recommendations for Future Research

The meta-analyses included in our meta-review varied considerably in terms of what was reported about the human support provided in the individual studies, making it challenging to see clear patterns in the results. To that end, we strongly recommend that future reports on meta-analyses and RCTs provide more detailed information. Our recommendations are similar to those made by Bernstein et al [39]. However, we suggest additional guidelines for reporting that do not exclusively focus on human support.

First, similar to Bernstein et al [39], we strongly recommend additional information about the training of individuals providing support (eg, therapists, graduate students, and paid research assistants). Although some meta-analyses made clear that they included studies that focused on only one type of supportive person (eg, clinician-supported interventions), most meta-analyses did not specify the training of the guidance or human support provider. Information about the type of training received by support providers is crucial, and future work should focus on including specific information about training and supervision (see the study by Werntz et al [76] for an example).

Second, in line with the study by Bernstein et al [39], researchers need to clearly define what the support providers are doing during the intervention. The studies included in these meta-analyses reported various types of supportive behaviors (both between and within meta-analyses). Although some meta-analyses included lists of the types of support behaviors, including writing emails to participants and texting to provide support, most did not provide that level of detail. We suspect that the kind of behavior expected from support providers largely influences the effectiveness of support. Until there is greater specificity, it will be difficult for the field to advance science-backed support guidelines.

Third, information about the DMHIs themselves needs to be included in the reports. In our meta-review, there was diversity in the types of interventions (mindfulness programs, cognitive behavioral programs, and cognitive bias modification) delivered as well as in the delivery approach (smartphone, CD-ROM, and internet). Interventions also varied widely in terms of recommended program length and the types of behaviors required by the user. We hypothesize that different types of support are needed for different types of programs; thus, additional information about the interventions needs to be more transparent for future investigations.

Finally, we recommend that researchers describe the mechanism through which they believe the coach will be most useful for the DMHI. For example, the supportive accountability model [35] posits that human support increases adherence to a DMHI, thereby increasing the efficacy of the intervention. However, other models of human support may combine supportive accountability with supervised practice of skills to transfer to the user’s real world [76], thereby increasing efficacy. As noted by Leung et al [52], there is considerable heterogeneity across studies in models of support. In the studies they analyzed, Leung et al [52] found that although most used a supportive accountability model, at least 1 study included support providers that focused on sharing their own experiences of recovery [77], suggesting a very different hypothesized mechanism for how human support may enhance DMHI efficacy. Bernstein et al [39] described a similar concept of testing hypothesized targets of coaching interventions. Understanding how human support increases the efficacy of interventions is a crucial next step in this field to fully leverage the potential of DMHIs.

### Conclusions

Mental health challenges and their associated impairments remain widespread and burdensome, particularly among individuals from culturally disadvantaged populations [78]. DMHIs offer promise of access to high-fidelity evidence-based interventions, and human support allows DMHI users to benefit from assistance and accountability. The findings of this meta-review suggest that human-supported DMHIs are a promising way to improve the impact of DMHIs on a range of mental health symptoms, and the human support does not have to come from highly trained mental health professionals. The combination of paraprofessional coaching and evidence-based DMHIs could bridge some of the current gaps in global mental health care. Future research will allow for an understanding of how models of human support can be matched to individuals’ backgrounds and types of DMHIs.

## Appendix

Multimedia Appendix 1 Articles excluded at the full-text level.

Multimedia Appendix 2 Assessment of Multiple Systematic Reviews 2 percentages achieved for each meta-analysis included.

## Abbreviations

AMSTAR: Assessment of Multiple Systematic Reviews
CCA: corrected covered area
DMHI: digital mental health intervention
MHA: mental health mobile app
PRISMA: Preferred Reporting Items for Systematic Reviews and Meta-Analyses
PTSD: posttraumatic stress disorder
RCT: randomized controlled trial

## Data Availability

Data are available upon request to the first author (AW).
