# Supplementary material for: Providing Human Support for the Use of Digital Mental Health Interventions: Systematic Meta-review
Source: J Med Internet Res. 2023 Feb 6;25:e42864. doi: 10.2196/42864 (PMC9941905; doi:10.2196/42864)
Supplement: Multimedia Appendix 1 [file jmir_v25i1e42864_app1.docx]

**Multimedia Appendix 1.** Articles excluded at full-text level

| Title | Authors | Year |
| --- | --- | --- |
| Cognitive behavior therapy for health anxiety: systematic review and meta-analysis of clinical efficacy and health economic outcomes | Axelsson, E., Hedman-Lagerlöf, E. | 2019 |
| Effect of cognitive-behavioral therapy for anxiety disorders on quality of life: a meta-analysis | Hofmann, S.G., Wu, J.Q., Boettcher, H. | 2014 |
| Smartphone-Based Interventions and Internalizing Disorders in Youth: Systematic Review and Meta-analysis. | Buttazzoni, A., Brar, K., Minaker, L. | 2021 |
| Internet Interventions for Adults with Anxiety and Mood Disorders: A Narrative Umbrella Review of Recent Meta-Analyses. | Andersson, G., Carlbring, P., Titov, N., Lindefors, N. | 2019 |
| The Acceptability and Usability of Digital Health Interventions for Adults With Depression, Anxiety, and Somatoform Disorders: Qualitative Systematic Review and Meta-Synthesis. | Patel, S., Akhtar, A., Malins, S., Wright, N., Rowley, E., Young, E., Sampson, S., Morriss, R. | 2020 |
| Internet- and Mobile-Based Interventions for Mental and Somatic Conditions in Children and Adolescents. | Domhardt, M., Steubl, L., Baumeister, H. | 2018 |
| Effects of Internet-Based Cognitive Behavioral Therapy in Routine Care for Adults in Treatment for Depression and Anxiety: Systematic Review and Meta-Analysis. | Etzelmueller, A., Vis, C., Karyotaki, E., Baumeister, H., Titov, N., Berking, M., Cuijpers, P., Riper, H., Ebert, D.D. | 2020 |
| Internet-delivered psychological interventions for clinical anxiety and depression in perinatal women: a systematic review and meta-analysis. | Loughnan, S.A., Joubert, A.E., Grierson, A., Andrews, G., Newby, J.M. | 2019 |
| Internet-based cognitive and behavioural therapies for post-traumatic stress disorder (PTSD) in adults. | Simon, N., Robertson, L., Lewis, C., Roberts, N.P., Bethell, A., Dawson, S., Bisson, J.I. | 2021 |
| Effectiveness of eHealth Interventions to Reduce Perinatal Anxiety: A Systematic Review and Meta-Analysis. | Bayrampour, H., Trieu, J., Tharmaratnam, T. | 2019 |
| Effect of smartphone app on post-traumatic stress disorder in COVID-19 convalescent patients: A protocol for systematic review and meta-analysis. | Wang, Y., Yang, X., Chen, H., Xu, Y. | 2021 |
| Digital Interventions for Screening and Treating Common Mental Disorders or Symptoms of Common Mental Illness in Adults: Systematic Review and Meta-analysis. | Sin, J., Galeazzi, G., McGregor, E., Collom, J., Taylor, A., Barrett, B., Lawrence, V., Henderson, C. | 2020 |
| E-Health interventions for anxiety and depression in children and adolescents with long-term physical conditions. | Thabrew, H., Stasiak, K., Hetrick, S.E., Wong, S., Huss, J.H., Merry, S.N. | 2018 |
| The effectiveness and cost-effectiveness of e-health interventions for depression and anxiety in primary care: A systematic review and meta-analysis. | Massoudi, B., Holvast, F., Bockting, C.L.H., Burger, H., Blanker, M.H. | 2019 |
| Internet-delivered cognitive behavioral therapies for late-life depressive symptoms: a systematic review and meta-analysis. | Xiang, X., Wu, S., Zuverink, A., Tomasino, K.N., An, R., Himle, J.A. | 2019 |
| Smartphone applications for the treatment of depressive symptoms: A meta-analysis and qualitative review. | Park, C., Zhu, J., Ho, Chun Man, R., Rosenblat, J.D., Iacobucci, M., Gill, H., Mansur, R.B., McIntyre, R.S. | 2020 |
| The efficacy of mindfulness meditation apps in enhancing users' well-being and mental health related outcomes: a meta-analysis of randomized controlled trials. | Gál, É., Ștefan, S., Cristea, I.A. | 2021 |
| The effectiveness of Internet-delivered treatment for generalized anxiety disorder: An updated systematic review and meta-analysis. | Eilert, N., Enrique, A., Wogan, R., Mooney, O., Timulak, L., Richards, D. | 2020 |
| Is self-guided internet-based cognitive behavioural therapy (iCBT) harmful? An individual participant data meta-analysis. | Karyotaki, E., Kemmeren, L., Riper, H., Twisk, J., Hoogendoorn, A., Kleiboer, A., Mira, A., Mackinnon, A., Meyer, B., Botella, C., Littlewood, E., Andersson, G., Christensen, H., Klein, J.P., Schröder, J., Bretón-López, J., Scheider, J., Griffiths, K., Farrer, L., Huibers, M.J.H., Phillips, R., Gilbody, S., Moritz, S., Berger, T., Pop, V., Spek, V., Cuijpers, P. | 2018 |
| Effectiveness of cognitive behavioural therapy for perinatal depression: A systematic review and meta-analysis. | Li, Z., Liu, Y., Wang, J., Liu, J., Zhang, C., Liu, Y. | 2020 |
| Theoretical adequacy, methodological quality and efficacy of online interventions targeting resilience: a systematic review and meta-analysis. | Díaz-García, A., Franke, M., Herrero, R., Ebert, D.D., Botella, C. | 2021 |
| Effectiveness of mindfulness-based intervention on psychotic symptoms for patients with schizophrenia: A meta-analysis of randomized controlled trials. | Liu, Y.C., Li, I.L., Hsiao, F.H. | 2021 |
| A Systematic Review of the Evidence Supporting Mobile- and Internet-Based Psychological Interventions For Self-Harm. | Arshad, U., Farhat-Ul-Ain, Gauntlett, J., Husain, N., Chaudhry, N., Taylor, P.J. | 2019 |
| Remote cognitive behavioral therapy for panic disorder: A meta-analysis. | Efron, G., Wootton, B.M. | 2021 |
| Internet-Delivered Cognitive Behavioral Therapy for Anxiety Disorders in Open Community Versus Clinical Service Recruitment: Meta-Analysis. | Romijn, G., Batelaan, N., Kok, R., Koning, J., van Balkom, A., Titov, N., Riper, H. | 2019 |
| Web-Based Interventions to Improve Mental Health in Home Caregivers of People With Dementia: Meta-Analysis. | Zhao, Y., Feng, H., Hu, M., Hu, H., Li, H., Ning, H., Chen, H., Liao, L., Peng, L. | 2019 |
| Annual Research Review: Digital health interventions for children and young people with mental health problems - a systematic and meta-review. | Hollis C; Falconer CJ; Martin JL; Whittington C; Stockton S; Glazebrook C; Davies EB | 2016 |
| Effectiveness and Safety of Using Chatbots to Improve Mental Health: Systematic Review and Meta-Analysis. | Abd-Alrazaq, A.A., Rababeh, A., Alajlani, M., Bewick, B.M., Househ, M. | 2020 |
| Internet-delivered cognitive behavioural therapy for post-traumatic stress disorder: systematic review and meta-analysis. | Lewis, C., Roberts, N.P., Simon, N., Bethell, A., Bisson, J.I. | 2019 |
| Are Internet- and mobile-based interventions effective in adults with diagnosed panic disorder and/or agoraphobia? A systematic review and meta-analysis. | Domhardt, M., Letsch, J., Kybelka, J., Koenigbauer, J., Doebler, P., Baumeister, H. | 2020 |
| Mobile Apps for Mental Health Issues: Meta-Review of Meta-Analyses. | Lecomte, T., Potvin, S., Corbière, M., Guay, S., Samson, C., Cloutier, B., Francoeur, A., Pennou, A., Khazaal, Y. | 2020 |
| Internet-Based Cognitive Behavioral Therapy to Reduce Suicidal Ideation: A Systematic Review and Meta-analysis. | Büscher, R., Torok, M., Terhorst, Y., Sander, L. | 2020 |
| Impact of mobile health interventions during the perinatal period on maternal psychosocial outcomes: a systematic review. | Dol, J., Richardson, B., Murphy, G.T., Aston, M., McMillan, D., Campbell-Yeo, M. | 2020 |
| Computerised therapies for anxiety and depression in children and young people: a systematic review and meta-analysis. | Pennant, M.E., Loucas, C.E., Whittington, C., Creswell, C., Fonagy, P., Fuggle, P., Kelvin, R., Naqvi, S., Stockton, S., Kendall, T. | 2015 |
| Internet-Based Supportive Interventions for Family Caregivers of People With Dementia: Systematic Review and Meta-Analysis. | Leng, M., Zhao, Y., Xiao, H., Li, C., Wang, Z. | 2020 |
| Efficacy and acceptability of mindfulness-based interventions for military veterans: A systematic review and meta-analysis. | Goldberg, S.B., Riordan, K.M., Sun, S., Kearney, D.J., Simpson, T.L. | 2020 |
| Effectiveness of a freely available computerised cognitive behavioural therapy programme (MoodGYM) for depression: Meta-analysis. | Twomey, C., O'Reilly, G. | 2016 |
| A systematic review and meta-analysis on the efficacy of Internet-delivered behavioral activation. | Huguet, A., Miller, A., Kisely, S., Rao, S., Saadat, N., McGrath, P.J. | 2018 |
| Internet-delivered psychological treatments for mood and anxiety disorders: a systematic review of their efficacy, safety, and cost-effectiveness. | Arnberg, F.K., Linton, S.J., Hultcrantz, M., Heintz, E., Jonsson, U. | 2014 |
| Evaluation of Technology-Based Interventions for Informal Caregivers of Patients With Dementia-A Meta-Analysis of Randomized Controlled Trials. | Deeken, F., Rezo, A., Hinz, M., Discher, R., Rapp, M.A. | 2019 |
| The effectiveness of the Internet-based self-management program for cancer-related fatigue patients: a systematic review and meta-analysis. | Huang, J., Han, Y., Wei, J., Liu, X., Du, Y., Yang, L., Li, Y., Yao, W., Wang, R. | 2020 |
| Impact of Internet-Based Interventions on Caregiver Mental Health: Systematic Review and Meta-Analysis. | Sherifali, D., Ali, MU., Ploeg, J., Markle-Reid, M., Valaitis, R., Bartholomew, A., Fitzpatrick-Lewis, D., McAiney, C. | 2018 |
| Suicide prevention using self-guided digital interventions: a systematic review and meta-analysis of randomised controlled trials. | Torok, M., Han, J., Baker, S., Werner-Seidler, A., Wong, I., Larsen, M.E., Christensen, H. | 2020 |
| Is cognitive behavioural therapy effective in reducing suicidal ideation and behaviour when delivered face-to-face or via e-health? A systematic review and meta-analysis. | Leavey, K., Hawkins, R. | 2017 |
| A meta-review of Internet computer-based psychological treatments for anxiety disorders. | Peñate, W., Fumero, A. | 2015 |
| Internet-supported versus face-to-face cognitive behavior therapy for depression. | Andersson, G., Topooco, N., Havik, O., Nordgreen, T. | 2015 |
| Effectiveness of online and mobile telephone applications ('apps') for the self-management of suicidal ideation and self-harm: a systematic review and meta-analysis. | Witt, K., Spittal, M.J., Carter, G., Pirkis, J., Hetrick, S., Currier, D., Robinson, J., Milner, A. | 2017 |
| Internet-assisted delivery of cognitive behavioural therapy (CBT) for childhood anxiety: systematic review and meta-analysis. | Rooksby, M., Elouafkaoui, P., Humphris, G., Clarkson, J., Freeman, R. | 2014 |
| eHealth interventions for the prevention of depression and anxiety in the general population: a systematic review and meta-analysis. | Deady, M., Choi, I., Calvo, R.A., Glozier, N., Christensen, H., Harvey, S.B. | 2017 |
| Outcomes associated with virtual reality in psychological interventions: where are we now? | Turner, W.A., Casey, L.M. | 2014 |
| Dismantling, optimising, and personalising internet cognitive behavioural therapy for depression: A systematic review and component network meta-analysis using individual participant data. | Furukawa, T. A., Suganuma, A., Ostinelli, E,G., Andersson, G., Beevers, C,G., Shumake, J., Berger, T., Boele, F.W., Buntrock, C., Carlbring, P., Choi, I., Christensen, H., Mackinnon, A., Dahne, J., Huibers, M.J.H., Ebert, D.D., Farrer, L., Forand, N.R., Strunk, D.R., Ezawa, I.D., Forsell, E., Kaldo, V., Geraedts, A., Gilbody, S., Littlewood, E., Brabyn, S., Hadjistavropoulos, H.D., Schneider, L.H., Johansson, R., Kenter, R., Kivi, M., Björkelund, C., Kleiboer, A., Riper, H., Klein, J.P., Schröder, J., Meyer, B., Moritz, S., Bücker, L., Lintvedt, O., Johansson, P., Lundgren, J., Milgrom, J., Gemmill, A.W., Mohr, D.C., Montero-Marin, J., Garcia-Campayo, J., Nobis, S., Zarski, A., O'Moore, K., Williams, A.D., Newby, J.M., Perini, S., Phillips, R., Schneider, J., Pots, W., Pugh, N.E., Richards, D., Rosso, I.M., Rauch, S.L., Sheeber, L.B., Smith, J., Spek, V., Pop, V.J., Ünlü, B., van Bastelaar, K.M.P.; van Luenen, S., Garnefski, N., Kraaij, V., Vernmark, K., Warmerdam, L., van Straten, A., Zagorscak, P., Knaevelsrud, C., Heinrich, M., Miguel, C., Cipriani, A., Efthimiou, O., Karyotaki, E., Cuijpers, P. | 2021 |
| Internet‐delivered cognitive behavioural therapy for post‐traumatic stress disorder: Systematic review and meta‐analysis. | Lewis, C., Roberts, N.P., Simon, N., Bethell, A., Bisson, J.I. | 2019 |
| Computer-delivered and web-based interventions to improve depression, anxiety, and psychological well-being of university students: A systematic review and meta-analysis. | Davies, E.B., Morriss, R., Glazebrook, C, | 2014 |
| Toward the design of evidence-based mental health information systems for people with depression: A systematic literature review and meta-analysis. | Wahle, F., Bollhalder, L., Kowatsch, T., Fleisch, E. | 2017 |
| Meta-analysis of technology-enabled mindfulness-based programs for negative affect and mindful awareness. | Victorson, D.E., Sauer, C.M., Wolters, L., Maletich, C., Lukoff, K., Sufrin, N. | 2020 |
| Internet-based cognitive behavioral therapy for insomnia (ICBT-i) improves comorbid anxiety and depression—A meta-analysis of randomized controlled trials. | Ye, Y., Zhang, Y., Chen, J., Liu, J., Li, X., Liu, Y., Lang, Y., Lin, L., Yang, X., Jiang, X. | 2015 |
| The efficacy of multi-component positive psychology interventions: A systematic review and meta-analysis of randomized controlled trials. | Hendriks, T., Schotanus-Dijkstra, M., Hassankhan, A., de Jong, J., Bohlmeijer, E. | 2020 |
| Impact of the method of delivering electronic health behavior change interventions in survivors of cancer on engagement, health behaviors, and health outcomes: Systematic Review and meta-analysis. | Furness, K., Sarkies, M.N., Huggins, C.E., Croagh, D., Haines, T.P. | 2020 |
| Response and remission rates in internet-based cognitive behavior therapy: An individual patient data meta-analysis. | Andersson, G., Carlbring, P., Rozental, A. | 2019 |
| Psychological treatments for depression delivered via the internet and supported by a clinician: An update. | Andersson, G., Nordgren, L.B., Buhrman, M., Carlbring, P. | 2014 |
| Task-sharing interventions for patients with anorexia nervosa or their carers: A systematic evaluation of the literature and meta-analysis of outcomes. | Albano, G., Hodsoll, J., Kan, C., Lo Coco G., Cardi, V. | 2019 |
| The effectiveness of various computer-based interventions for patients with chronic pain or functional somatic syndromes: A systematic review and meta-analysis. | Vugts, M.A.P., Joosen, M.C.W., van der Geer, J.E., Zedlitz, A.M.E.E., Vrijhoef, H.J.M. | 2018 |
| Tailored web-based interventions for pain: Systematic review and meta-analysis. | Martorella, G., Boitor, M., Berube, M., Fredericks, S., Le May, S., Gélinas, C. | 2017 |
| Internet-delivered cognitive behavior therapy for children and adolescents: A systematic review and meta-analysis. | Vigerland, S., Lenhard, F., Bonnert, M., Lalouni, M., Hedman, E., Ahlen, J., Olén, O., Serlachius, E., Ljótsson, B. | 2016 |
| Do Web-based interventions improve well-being in type 2 diabetes? A systematic review and meta-analysis. | Hadjiconstantinou, M., Byrne, J., Bodicoat, D.H., Robertson, N., Eborall, Helen., Khunti, K., Davies, M. | 2016 |
| For better or worse: An individual patient data meta-analysis of deterioration among participants receiving Internet-based cognitive behavior therapy. | Rozental, A., Magnusson, K., Boettcher, J., Andersson, G., Carlbring, P. | 2017 |
| Mobile technology boosts the effectiveness of psychotherapy and behavioral interventions: A meta-analysis. | Lindhiem, O., Bennett, C.B., Rosen, D., Silk, J. | 2015 |
| Internet-based cognitive behavioral therapy for patients with chronic somatic conditions: A meta-analytic review. | van Beugen, S., Ferwerda, M., Hoeve, D., Rovers, M.M., Koulil, S.S., van Middendorp, H., Evers, A.W.M. | 2014 |
| An evaluation of the effectiveness of the modalities used to deliver electronic health interventions for chronic pain: Systematic review with network meta-analysis. | Slattery, B.W., Haugh, S., O'Connor, L., Francis, K., Dwyer, C.P., O'Higgins, S., Egan, J., McGuire, B.E. | 2019 |
| A review and meta-analysis of perfectionism interventions: Comparing face-to-face with online modalities. | Suh, H., Sohn, H., Kim, T., Lee, D. | 2019 |
| Internet-based prenatal interventions for maternal health among pregnant women: A systematic review and meta-analysis. | Chae, J., Kim, H.K. | 2021 |
| Low intensity technology-delivered cognitive behavioral therapy for obsessive-compulsive disorder: A meta-analysis. | Hoppen, L.M., Kuck, N., Bürkner, P., Karin, E., Wootton, B.M., Buhlmann, U. | 2021 |
| Effects of cognitive bias modification on social anxiety: A meta-analysis. | Liu, H., Li, X., Han, B., Liu, X. | 2017 |
| Digital health behaviour change interventions targeting physical activity and diet in cancer survivors: A systematic review and meta-analysis. | Roberts, A.L., Fisher, A., Smith, L., Heinrich, M., Potts, H.W.W. | 2017 |
| Treatment of comorbid alcohol use disorders and depression with cognitive‐behavioural therapy and motivational interviewing: A meta‐analysis. | Riper, H., Andersson, G., Hunter, S.B., de Wit, J., Berking, M., Cuijpers, P. | 2014 |
| Self-management intervention for adult cancer survivors after treatment: A systematic review and meta-analysis. | Kim, SH., Kim, K., Mayer, D.K. | 2017 |
| Influence of initial severity of depression on effectiveness of low intensity interventions: Meta-analysis of individual patient data. | Bower, P., Kontopantelis, E., Sutton, A., Kendrick, T., Richards, D.A., Gilbody, S., Knowles, S., Cuijpers, P., Andersson, G., Christensen, H., Meyer, B., Huibers, M., Smit, F., van Straten, A., Warmerdam, L., Barkham, M., Bilich, L., Lovell, K., Liu, E.T. | 2013 |
| Do guided internet-based interventions result in clinically relevant changes for patients with depression? An individual participant data meta-analysis. | Karyotaki, E., Ebert, D.D., Donkin, L., Riper, H., Twisk, J., Burger, S., Rozental, A., Lange, A., Williams, A.D., Zarski, A.C., Geraedts, A., van Straten, A., Kleiboer, A., Meyer, B., Ünlü Ince, B.B., Buntrock, C., Lehr, Dirk., Snoek, F.J., Andrews, G., Andersson, G., Choi, I., Ruwaard, J., Klein, J.P., Newby, J.M., Schröder, J., Laferton, J.A.C., Van Bastelaar, K., Imamura, K., Vernmark, K., Boß, L., Sheeber, L.B., Kivi, M., Berking, M., Titov, N., Carlbring, P., Johansson, R., Kenter, R., Perini, S., Moritz, S., Nobis, S., Berger, T., Kaldo, V., Forsell, Y., Lindefors, N., Kraepelien, M., Björkelund, C., Kawakami, N., Cuijpers, P. | 2018 |
| Psychological interventions for the fear of public speaking: A meta-analysis. | Ebrahimi, O.V., Pallesen, S., Kenter, R.M.F., Nordgreen, T. | 2019 |
| Adolescent sleep: Comorbid problems and behavioral treatment of insomnia. | Åslund, L. | 2020 |
| Do depressive symptoms affect the outcome of treatments for SAD? A meta analysis of randomized controlled trials. | Rozen, N., Aderka, I.M. | 2020 |
| In the absence of effects: An individual patient data meta-analysis of non-response and its predictors in internet-based cognitive behavior therapy. | Rozental, A., Andersson, G., Carlbring, P. | 2019 |
| Effect of music therapy on pain after orthopedic surgery—A systematic review and meta‐analysis. | Lin, C., Hwang, S., Jiang, P., Hsiung, N. | 2020 |
| Internet-delivered cognitive behavioral therapy for panic disorder with or without agoraphobia: A systematic review and meta-analysis. | Stech, E.P., Lim, J., Upton, E.L., Newby, J.M. | 2020 |
| Efficacy of internet-delivered psychodynamic therapy: Systematic review and meta-analysis. | Lindegaard, T., Berg, M., Andersson, G. | 2020 |
| Efficacy, acceptability and safety of internet‐delivered psychological therapies for fibromyalgia syndrome: A systematic review and meta‐analysis of randomized controlled trials. | Bernardy, K., Klose, P., Welsch, P., Häuser, W. | 2018 |
| Computer therapy for the anxiety and depression disorders is effective, acceptable and practical health care: An updated meta analysis | G. Andrews, A. Basu, P. Cuijpers, M.G. Craske, P. McEvoy, C.L. English, J.M. Newby | 2018 |
| Digital Parent Training for Children with Disruptive Behaviors: Systematic Review and Meta-Analysis of Randomized Trials | Amit B., Aditya P., John M.K., Christoph U.C. | 2016 |
| eHealth to Redress Psychotherapy Access Barriers Both New and Old: A Review of Reviews and Meta-Analyses | Charles B. Bennett, Camilo J. Ruggero, Anna C. Sever, Lamia Yanouri | 2020 |
| Internet-based treatment of social phobia: A randomized controlled trial comparing unguided with two types of guided self-help | Berger, T., Caspar, F., Richardson, R., Kneubühler, B., Sutter, D., Andersson, G. | 2011 |
| Effectiveness of Web-Delivered Acceptance and Commitment Therapy in Relation to Mental Health and Well-Being: A Systematic Review and Meta-Analysis | Brown, M., Glendenning, A., Hoon, A.E., John, A. | 2016 |
| Internet-based vs. face-to-face cognitive behavior therapy for psychiatric and somatic disorders: an updated systematic review and meta-analysis | Carlbring, P., Andersson, G., Cuijpers, P., Riper, H., Hedman-Lagerlöf, E. | 2018 |
| Computer-Delivered and Web-Based Interventions to Improve Depression, Anxiety, and Psychological Well-Being of University Students: A Systematic Review and Meta-Analysis | Davies, E.B., Morriss, R., Glazebrook, C. | 2014 |
| eHealth interventions for the prevention of depression and anxiety in the general population: a systematic review and meta-analysis | Deady, M., Choi, I., Calvo, R.A., Glozier, N., Christensen, H., Harvey, S.B. | 2017 |
| Internet and Computer-Based Cognitive Behavioral Therapy for Anxiety and Depression in Youth: A Meta-Analysis of Randomized Controlled Outcome Trials | Ebert, D.D., Zarski, A., Christensen, H., Stikkelbroek, Y., Cuijpers, P., Berking, M., Riper, H. | 2015 |
| Does Internet-based guided-self-help for depression cause harm? An individual participant data meta-analysis on deterioration rates and its moderators in randomized controlled trials | Ebert, DD., Donkin, L., Andersson, G., Andrews, G., Berger, T., Carlbring, P., Rozenthal, A., Choi, I., Laferton, J.A.C., Johansson, R., Kleiboer, A., Lange, A., Lehr, D., Reins, J.A. Funk, B., Newby, J., Perini, S., Riper, H., Ruwaard, J., Sheeber, L., Snoek, F.J., Titov, N., Ünlü Ince, B., van Bastelaar, K., Vernmark, K., van Straten, A., Warmerdam, L., Salsman, N., Cuijpers, P. | 2016 |
| Can smartphone mental health interventions reduce symptoms of anxiety? A meta-analysis of randomized controlled trial | Firth, J., Torous, J., Nicholas, J., Carney, R., Rosenbaum, S., Sarris, J. | 2017 |
| The efficacy of smartphone-based mental health interventions for depressive symptoms: a meta-analysis of randomized controlled trials | Firth, J., Torous, J., Nicholas, J., Carney, R., Pratap, A., Rosenbaum, S., Sarris, J. | 2017 |
| Guided Internet-Based Self-Help Intervention for Social Anxiety Disorder With Videoconferenced Therapist Support | Gershkovich, M., Herbert, J. D., Forman, E. M., Glassman, L. | 2016 |
| Internet- and mobile-based depression interventions for people with diagnosed depression: A systematic review and meta-analysis | Josephine, K., Josefine, L., Philipp, D., David, E., Harald, B. | 2017 |
| Efficacy of Self-guided Internet-Based Cognitive Behavioral Therapy in the Treatment of Depressive Symptoms: A Meta-analysis of Individual Participant Data | Karyotaki, Riper, H., Twisk, J., Hoogendoorn, A., Kleiboer, A., Mira, A., Mackinnon, A., Meyer, B., Botella, C., Littlewood, E., Andersson, G., Christensen, H., Klein, J.P., Schröder, J., Bretón-López, J., Scheider, J., Griffiths, K., Farrer, L., Huibers, M.J.H., Phillips, R., Gilbody, S., Moritz, S., Berger, T., Pop., Spek., Cuijpers, P. | 2017 |
| Do guided internet-based interventions result in clinically relevant changes for patients with depression? An individual participant data meta-analysis | Karyotaki, Ebert, D. D., Donkin, L., Riper, H., Twisk, J., Burger, S., Rozental, A., Lange, A., Williams, A. D., Zarski, A. C., Geraedts, A., van Straten, A., Kleiboer, A., Meyer, B., Ünlü Ince, B. B., Buntrock, C., Lehr, D., Snoek, F. J., Andrews, G., Andersson, G., Choi, I., Ruwaard, J., Klein, J. P., Newby, J.M., Schröder, J., Laferton, J.A.C., Van Bastelaar, K., Imamura, K., Vernmark, K., Boß, L., Sheeber, L.B., Kivi, M., Berking, M., Titov, N., Carlbring, P., Johansson, R., Kenter, R., Perini, S., Moritz, S., Nobis, S., Berger, T., Kaldo, V., Forsell, Y., Lindefors, N., Kraepelien, M., Björkelund, C., Kawakami, N., Cuijpers, P. | 2018 |
| Therapist-Supported Internet-Based Cognitive Behavior Therapy for Stress, Anxiety, and Depressive Symptoms Among Postpartum Women:A Systematic Review and Meta-Analysis | Lau, Y., Htun, T.P., Wong, S.N., Tam, W.S.W., Klainin-Yobas, P. | 2017 |
| Transdiagnostic computerised cognitive behavioural therapy for depression and anxiety: A systematic review and meta-analysis | Newby, J.M., Twomey, C., Yuan Li, S.S., Andrews, G. | 2016 |
| The Efficacy and Acceptability of Third-Wave Behavioral and Cognitive eHealth Treatments: A Systematic Review and Meta-Analysis of Randomized Controlled Trials | O’Connor, M., Munnelly, A., Whelan, R., McHugh, L. | 2018 |
| Internet-assisted delivery of cognitive behavioural therapy (CBT) for childhood anxiety: Systematic review and meta-analysis | Rooksby, M., Elouafkaoui, P., Humphris, G., Clarkson, J., Freeman, R. | 2014 |
| The Potential of Technology-Based Psychological Interventionsfor Anorexia and Bulimia Nervosa: A Systematic Review and Recommendations for Future Research | Schlegl, S., Bürger, C., Schmidt, L., Herbst, N., Voderholzer, U. | 2015 |
| eHealth and mHealth interventions in the treatment of fatigued cancer survivors: A systematic review and meta‐analysis | Seiler, A., Klaas, V., Tröster, G., Fagundes, C.P. | 2017 |
| Effectiveness of a freely available computerised cognitive behavioural therapy programme (MoodGYM) for depression: Meta-analysis | Twomey, C., O’Reilly, G. | 2017 |
| Web-Based Interventions Supporting Adolescents and Young People With Depressive Symptoms: Systematic Review and Meta-Analysis | Välimäki, M., Anttila, K., Anttila, M., Lahti, M. | 2017 |
| Internet-delivered cognitive behavior therapy for children and adolescents: A systematic review and meta-analysis | Vigerland, S., Lenhard, F., Bonnert, M., Lalouni, M., Hedman, E., Ahlen, J., Olén, O., Serlachius, E., Ljótsson, B. | 2016 |
| Effects of Internet-based psycho-educational interventions on mental health and quality of life among cancer patients: a systematic review and meta-analysis | Wang, Y., Lin, Y., Chen, J., Wang, C., Hu, R., Wu, Y. | 2020 |
| Standalone smartphone apps for mental health — a systematic review and meta-analysis | Weisel, K.K., Fuhrmann, L. M., Berking, M., Baumeister, H., Cuijpers, P., Ebert, D. D. | 2019 |
| Effectiveness of internet-based interventions for children, youth, and young adults with anxiety and/or depression: a systematic review and meta-analysis | Ye, X., Bapuji, S.B., Winters, S.E., Struthers, A., Raynard, M., Metge, C., Kreindler, S.A., Charette, C.J., Lemaire, J.A., Synyshyn, M., Sutherland, K. | 2014 |
| Internet-based cognitive behavioural therapy for subthreshold depression: a systematic review and meta-analysis | Zhou, T., Li, X., Pei, Y., Gao, J., Kong, J. | 2016 |
| Eﬀectiveness of an individually-tailored computerised CBT programme (Deprexis) for depression: A meta-analysis | Twomey, C., O’Reilly, G., Meyer, B. | 2017 |
| A meta-analysis: Internet mindfulness-based interventions for stress management in the general population | Zhang, Y., Xue, J., Huang, Y. | 2020 |
| Improving Employee Well-Being and Effectiveness: Systematic Review and Meta-Analysis of Web-Based Psychological Interventions Delivered in the Workplace. | Carolan, S., Harris, P. R., Cavanagh, K. | 2017 |
