# Supplementary material for: Providing Human Support for the Use of Digital Mental Health Interventions: Systematic Meta-review
Source: J Med Internet Res. 2023 Feb 6;25:e42864. doi: 10.2196/42864 (PMC9941905; doi:10.2196/42864)
Supplement: Multimedia Appendix 2 [file jmir_v25i1e42864_app2.docx]

**Multimedia Appendix 2.** AMSTAR 2 percentages achieved for each meta-analysis included

**Carolan et al, 2017:** 40.63

**Cheng, et al. 2020:** 43.75

**Conley et al., 2016:** 40.63

**Cowpertwait et al, 2013:** 31.25

**Domhardt et al, 2019:** 62.5

**Firth, et al., 2017:** 37.

**Fu, et al. (2020):** 65.63

**Grist et al., 2019:** 34.38

**Harrer et al, 2018:** 62.5

**Heber, et al., 2017:** 62.5

**Kampman et al, 2016:** 53.13

**Kuester, et al., 2016:** 43.75

**Li et al, 2014:** 53.13

**Linardon, et al., 2019:** 43.75

**​​Pang et al, 2021:** 65.63

**Păsărelu et al, 2017:** 43.75

**Phillips et al, 2019:** 62.5

**Richards & Richardson, 2012:** 34.38

**Sherifali et al, 2018:** 50

**Sijbrandij et al, 2016:** 28.13

**Simmonds-Buckley et al, 2020:** 65.63

**Spijkerman et al, 2016:** 46.88

**Stratton et al, 2017:** 25

**Swati et al, 2019:** 46.88

**Sztein et al., 2018:** 59.375

**Thompson et al, 2021:** 81.25

**Twomey et al, 2020:** 65.63

**Versluis et al, 2016:** 46.8

**Victorson et al, 2020:** 40.63

**Wright, 2019:** 46.63
